# Identification of P genome chromosomes in *Agropyron cristatum* and wheat-*A. cristatum* derivative lines by FISH

**DOI:** 10.1038/s41598-019-46197-6

**Published:** 2019-07-04

**Authors:** Haiming Han, Weihua Liu, Jinpeng Zhang, Shenghui Zhou, Xinming Yang, Xiuquan Li, Lihui Li

**Affiliations:** National Key Facility for Crop Gene Resources and Genetic Improvement (NKCRI), Institute of Crop Sciences, Chinese Academy of Agricultural Sciences, Beijing, 100081 China

**Keywords:** Cytogenetics, Plant breeding

## Abstract

*Agropyron cristatum* (L.) Gaertn. (P genome) is cultivated as pasture fodder and can provide many desirable genes for wheat improvement. With the development of genomics and fluorescence *in situ* hybridization (FISH) technology, probes for identifying plant chromosomes were also developed. However, there are few reports on *A. cristatum* chromosomes. Here, FISH with the repeated sequences pAcTRT1 and pAcpCR2 enabled the identification of all diploid *A. cristatum* chromosomes. An integrated idiogram of *A. cristatum* chromosomes was constructed based on the FISH patterns of five diploid *A. cristatum* individuals. Structural polymorphisms of homologous chromosomes were observed not only among different individuals but also within individuals. Moreover, seventeen wheat-*A. cristatum* introgression lines containing different P genome chromosomes were identified with pAcTRT1 and pAcpCR2 probes. The arrangement of chromosomes in diploid *A. cristatum* was determined by identifying correspondence between the P chromosomes in these genetically identified introgression lines and diploid *A. cristatum* chromosomes. The two probes were also effective for discriminating all chromosomes of tetraploid *A. cristatum*, and the differences between two tetraploid *A. cristatum* accessions were similar to the polymorphisms among individuals of diploid *A. cristatum*. Collectively, the results provide an effective means for chromosome identification and phylogenetic studies of P genome chromosomes.

## Introduction

Chromosome identification plays an important role in genomic relationships, flow sorting, and polyploidization. Understanding the homoeology of alien chromosomes with wheat chromosomes will facilitate the utilization of favourable genes on alien chromosomes for wheat improvement^1^. The formation of common wheat (*Triticum aestivum* L.) represents a classic allopolyploidization event, which involved many chromosomal structural changes^2^. Chromosome identification can reveal the phenomenon of ‘apparent aneuploidy’ and wheat structural variations that arise during the process of allopolyploidization^3,4^. Additionally, chromosome discrimination contributes to wheat sequencing. Wheat chromosomes are isolated and sequenced by means of flow-cytometric sorting, which discriminates chromosomes by size or labelling patterns of the repetitive DNA probes^5,6^.

Fluorescence *in situ* hybridization (FISH) is valuable for detecting chromosomal aberrations, defining the chromosomes involved in cases of aneuploidy, studying chromosomal behaviour and the genomic localization of repetitive DNA sequence arrays and individual loci^7^. FISH with genome-specific dispersed repetitive sequences as probes can identify alien chromosomes in wheat backgrounds and reveal their distributions on chromosomes. Related investigations have been conducted in members of Triticeae, such as rye, *Dasypyrum villosum*, and *Agropyron cristatum*^8–12^. FISH with the *Aegilops tauschii* Coss. clone pAs1 and the rye clone pSc119.2 or the barley clone pHvG38 allowed easy discrimination of the three genomes of wheat^13,14^. The combined use of these probes with cDNA probes can allow the detection of homoeology with and chromosomal rearrangements of wild relatives^15,16^. Recently, oligonucleotide probes developed based on repetitive sequences have been used to identify chromosomes of wheat and structural chromosomal rearrangements and polymorphisms in widely grown wheat cultivars and their founders^17–20^.

*Agropyron* Gaertn., a perennial genus of the tribe Triticeae, contains one basic P genome and exhibits three ploidy levels: diploid, tetraploid, and hexaploid^21^. *Agropyron* species have high economic value. Due to its high feed value and roles in the improvement of degenerated natural grassland and the construction of artificial pasture, *Agropyron* is regarded highly by agrostologists^22^. *Agropyron* Gaertn., an important genus of wild relatives of wheat, possesses many desirable traits for wheat improvement, such as resistance to certain diseases and abiotic stress as well as multiple spikelets, florets, and fertile tiller number^21,23^. In our laboratory, wide hybridization of the common wheat cultivar Fukuhokomugi (Fukuho) and tetraploid *A. cristatum* (accession Z559) was achieved via embryo rescue^24^. A series of wheat-*A. cristatum* derivative lines conferring many desirable traits were obtained, including wheat-*A. cristatum* addition lines, substitution lines, deletion lines, and translocation lines^25–35^. These lines have been applied as bridge materials or novel germplasms in wheat improvement. The identification of P chromosomes is important, and probes have been used for the characterization of *A. cristatum* chromosomes, such as 5S rDNA, 45S rDNA, Afa, pSc119.2 and pSc200^36,37^. Nevertheless, additional probes are required for the precise identification of the derivative lines containing *A. cristatum* chromatin.

The aim of this study was to identify P genome chromosomes using FISH with pAcTRT1 and pAcpCR2 as probes and to unveil the structural polymorphisms of diploid *A. cristatum* chromosomes. The system was applied to identify P genome chromosomes in wheat-*A. cristatum* introgression lines and tetraploid *A. cristatum*. The results provide insights into the *A. cristatum* chromosomes involved in alien gene introgression and into the evolution of *A. cristatum*.

## Results

### pAcTRT1 and pAcpCR2 allowed the discrimination of *A. cristatum* chromosomes

According to our previous results^12^, the FISH signals of pAcTRT1 and pAcpCR2 varied on different chromosomes of diploid *A. cristatum* (Supplementary Fig. 1a,c). The signals of pAcTRT1 distributed in the telomeric regions of chromosomes, and signal distribution and intensity varied among the chromosomes (Supplementary Fig. 1b). Two of the seven chromosome pairs were discriminated when pAcpCR2 was used as a probe: the short arms of one chromosome pair were full of fluorescence signals, whereas the short arms of another pair showed a discontinuous distribution of fluorescence signals (Supplementary Fig. 1d). These findings indicated that these two probes can be used for the identification of *A. cristatum* chromosomes.

### Construction of the idiogram for *A. cristatum* chromosomes

**Figure 1:**
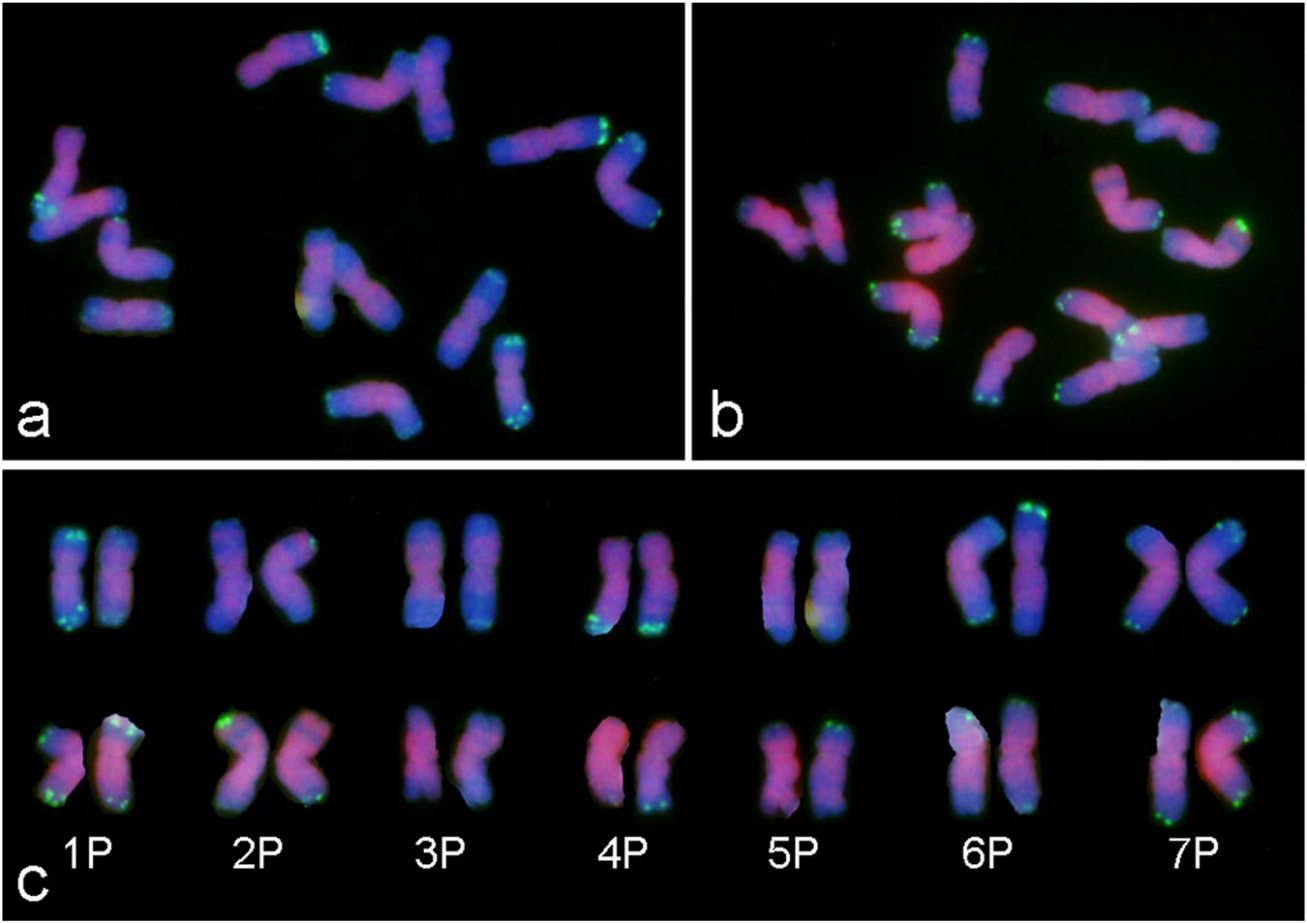
Comparison of P genome chromosomes on the basis of FISH patterns from two individual plants of diploid *A*. *cristatum* Z1842. The green signals represent the probe pAcTRT1, and the red signals represent the probe pAcpCR2.

The combination of pAcTRT1 and pAcpCR2 probes was used in FISH to identify the chromosomes in diploid *A*. *cristatum* (Fig. 1). The identification of *A*. *cristatum* chromosomes mainly depended on the FISH patterns, followed by relative chromosome lengths and arm ratios. The arrangement of *A*. *cristatum* chromosomes was determined based on subsequent analysis of the P chromosomes in genetically identified wheat-*A. cristatum* introgression lines. The relative arm lengths, relative chromosome lengths, and arm ratios (long/short) were measured (Table 1). All examined mitotic metaphase cells showed a standard diploid constitution (2*n* = 14), which was characterized by seven pairs of chromosomes. Subsequently, an idiogram showing the pAcTRT1 and pAcpCR2 signals was constructed.

**Table 1:** Chromosome parameters of the P genome.

Chromosome no.	Relative length of long arm^a^	Relative length of short arm^a^	Relative length of total chromosome	Arm ratio
1P	7.70 ± 0.40	4.98 ± 0.31	12.68 ± 0.38	1.55 ± 0.15
2P	8.90 ± 0.62	6.05 ± 0.41	14.95 ± 0.45	1.47 ± 0.19
3P	7.21 ± 0.30	6.53 ± 0.30	13.74 ± 0.48	1.10 ± 0.06
4P	8.38 ± 0.51	4.72 ± 0.031	13.10 ± 0.53	1.77 ± 0.18
5P	9.45 ± 0.54	4.62 ± 0.33	14.07 ± 0.63	2.05 ± 0.18
6P	8.35 ± 0.21	7.49 ± 0.27	15.84 ± 0.38	1.11 ± 0.04
7P	9.05 ± 0.39	6.83 ± 0.49	15.88 ± 0.69	1.32 ± 0.10

^a^Relative length = arm length/total length of haploid genome × 100. The data represent the mean (±standard deviation) of fifty cells.

**Figure 2:**
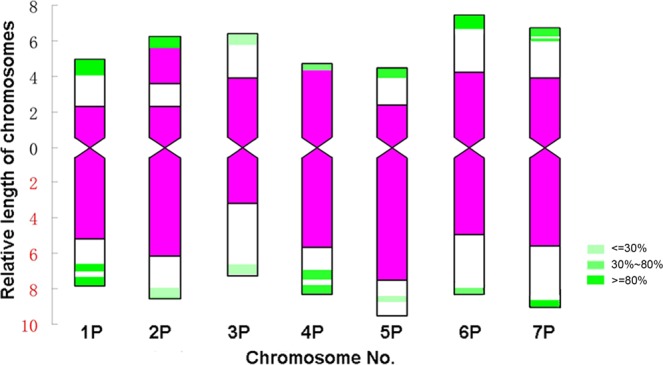
Integrated idiogram of *A. cristatum* chromosomes showing the locations of the pAcTRT1 and pAcpCR2 bands. The green signals represent the probe pAcTRT1, and the red signals represent the probe pAcpCR2. The colour gradient on the right shows the frequency of pAcTRT1 signals from five specimens of *A. cristatum*.

Comparison of the FISH patterns of different cells from five individuals revealed a diversity of pAcTRT1 signals in certain homologous chromosomes among different individuals as well as within individuals (Fig. 1). The diversity indicated the presence of chromosomal structural variations. To analyze the chromosomal polymorphisms of FISH patterns, all the FISH signals of the homologous chromosomes from the five individuals were combined, and a colour gradient was used to represent the different frequencies of FISH signals (Fig. 2). The integrated idiogram of *A. cristatum* chromosomes showed 17 pAcTRT1 signals, and these signals mainly distributed in the telomeric regions. The frequency of pAcTRT1 signals was 10% to 100%, with seven different gradients. The frequency of signals on chromosomes 1PS, 1PL, 4PL, and 7PS was 100%, and the lowest frequency of signals was observed for chromosome 3P.

The main features of pAcTRT1 and pAcpCR2 distributed on P genome chromosomes were as follows. The hybridization signals of pAcTRT1 on chromosome 1P were highly intense and stable on both arms. The signals generated by pAcpCR2 were discontinuous and divided into two sections on the short arm of 2P. Chromosome 3P had a small value of arm ratio and was an approximated metacentric chromosome. The area of signals generated by pAcpCR2 on the short arm was larger than that on the long arm, and the pAcTRT1 signals were weak. Chromosome 4P was characterized by pAcpCR2 signals covering the short arm and strong and stable pAcTRT1 signals on the long arm. Chromosome 5P displayed the largest value of arm ratio. Chromosome 6P was characterized by a small value of arm ratio and strong and stable signals on the short arm. The main feature of chromosome 7P was the two-band signals of pAcTRT1 on the short arm that were stronger than the signals on the long arm.

### pAcTRT1 and pAcpCR2 enabled the identification of P genome chromosomes in wheat-*A. cristatum* introgression lines

**Figure 3:**
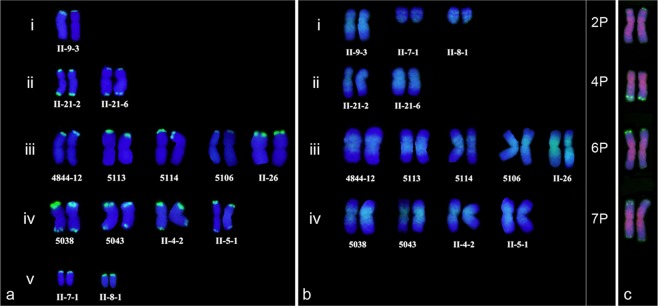
Classification of added chromosomes in fourteen wheat-*A. cristatum* addition lines identified by pAcTRT1 (**a**) and pAcpCR2 (**b**) and the corresponding chromosomes in diploid *A. cristatum* (**c**).

To explore the potential use of pAcTRT1 and pAcpCR2 in the identification of wheat*-A. cristatum* addition lines, FISH was performed to identify 14 different wheat*-A. cristatum* addition lines using pAcTRT1 and pAcpCR2 as probes. These lines were classified into four groups according to the signals generated by the pAcTRT1 probe and size of the P genome chromosomes (Fig. 3a, Supplementary Fig. 2). II-9-3 was included in group i, which was characterized by the presence of signals only on the short arm and by a large arm ratio. Group ii comprised II-21-2 and II-21-6 and was characterized by stronger signals on the long arm than on the short arm. 4844-12, 5113, 5114, 5106 and II-26 composed group iii, which displayed a small arm ratio and pAcTRT1 signals only on the short arm. 5038, 5043, II-4-2 and II-5-1, which composed group iv, had the common characteristic of stronger signals on the short arm than on the long arm. II-7-1 and II-8-1, two ditelosomic addition lines, displayed signals in the telomeric regions. With pAcpCR2, we determined that the P genome chromosome arms were the same as the short arm of II-9-3, characterized by discontinuous signals. Furthermore, the classification of II-21-2 and II-21-6 into one group characterized by short arms full of signals produced by probe pAcpCR2 were confirmed (Fig. 3b, Supplementary Fig. 3). The classification in the present study was consistent with our previous results that identified homoeologous groups of added *A. cristatum* chromosomes in these addition lines using molecular markers^27,38^.

**Figure 4:**
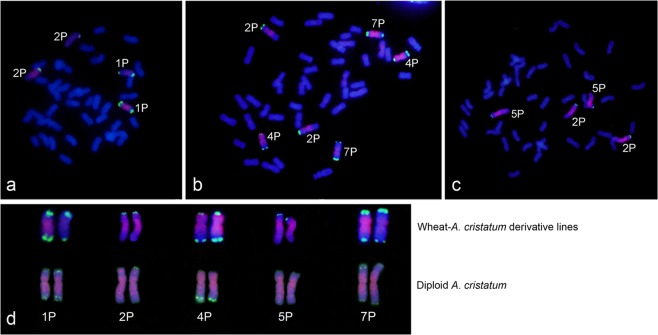
FISH patterns of wheat-*A. cristatum* derivative lines II-3-1 (**a**), II-23-1 (**b**) and II-11-1 (**c**) with multiple P genome chromosomes using pAcTRT1 and pAcpCR2 as probes and the corresponding chromosomes in diploid *A. cristatum* (**d**).

In addition, the wheat-*A. cristatum* derivatives II-3-1, II-23-1 and II-11-1 were identified by FISH using pAcTRT1 and pAcpCR2 as probes. We found that two, three and two different pairs of P genome chromosomes were present in II-3-1, II-23-1 and II-11-1, respectively; these pairs were determined to be 1P and 2P in II-3-1; 2P, 4P and 7P in II-23-1; and 2P and 5P in II-11-1 (Fig. 4) in our previous reports^34,38,39^.

### Classification of P genome chromosomes in diploid *A. cristatum*

The identification of P genome chromosomes in wheat-*A. cristatum* addition lines was informative for arranging the chromosomes of diploid *A. cristatum* corresponding to the homoeologous chromosomes of wheat. The P genome chromosomes in groups i, ii, iii, and iv were identified as 2P, 4P, 6P, and 7P, respectively (Fig. 3), in our previous studies^27,38^. According to the FISH patterns obtained using pAcTRT1 and pAcpCR2 as probes, we determined the corresponding chromosomes in diploid *A. cristatum* (Fig. 3c). Similarly, the chromosomes 1P and 5P were determined based on the FISH patterns of II-3-1 and II-11-1 (Fig. 4a,c). Then, the remaining chromosome in diploid *A. cristatum* was identified as 3P. All the arrangements of the P genome chromosomes in diploid *A. cristatum*, from 1P to 7P, were performed according to this system (Figs 1, 2).

**Figure 5:**
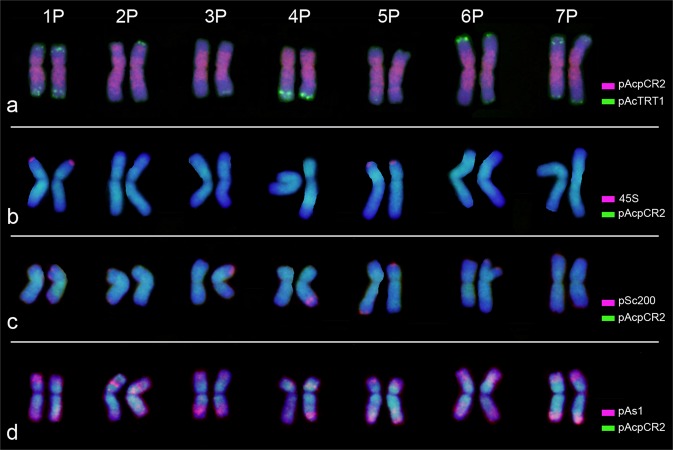
FISH performed on mitotic chromosomes of diploid *A. cristatum* Z1842 with probes for pAcTRT1, pAcpCR2, 45S rDNA, rye tandem pSc200 and pAs1.

Additionally, FISH with pAcpCR2 and 45S rDNA or rye tandem pSc200 repeat or pAs1 as probes was performed on mitotic chromosomes of diploid *A. cristatum* Z1842. The 45S rDNA probe showed signals on chromosomes 1P and 5P. pSc200 was found to produce signals on chromosomes 1P-7P, and polymorphisms occurred between homologous chromosomes except for chromosomes 1P and 7P (Fig. 5, Supplementary Fig. 4). Although the distribution of pSc200 was different from that of pAcTRT1, pSc200 together with pAcpCR2 could discriminate the P genome chromosomes. The pAs1 signals on P genome chromosomes appeared mainly in terminal and subterminal positions in accession Z1842, which also could discriminate the P genome chromosomes. The combined use of pAcpCR2 and 45S rDNA probes or pAcpCR2 and pSc200 probes or pAcpCR2 and pAs1 probes confirmed the order of P genome chromosomes through comparison of the FISH patterns of these probe combinations (Fig. 5).

### Comparison of the FISH patterns of two tetraploid *A. cristatum* accessions

**Figure 6:**
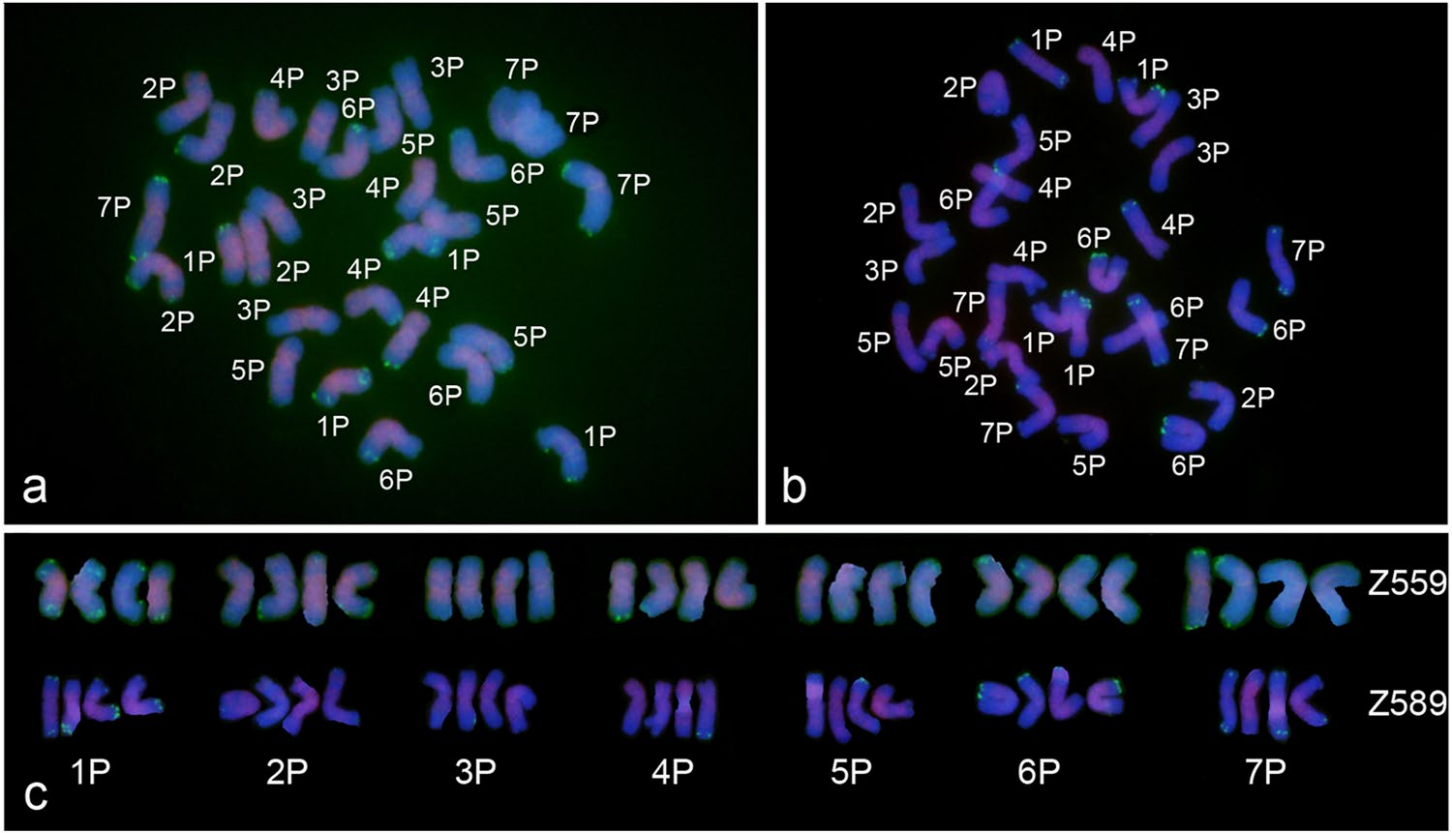
FISH patterns of the two tetraploid *A. cristatum* accessions Z559 (**a**) and Z589 (**b**) and a comparison of FISH patterns of these accessions (**c**). The green signals represent the probe pAcTRT1, and the red signals represent the probe pAcpCR2.

To verify the feasibility of chromosome identification in tetraploid *A. cristatum*, the probes pAcTRT1 and pAcpCR2 were used to identify chromosomes of tetraploid *A. cristatum* accessions Z559 and Z589, which were collected from Xinjiang and Inner Mongolia, China, respectively. The results indicated that these probes distributed differently among the different chromosomes and could discriminate all the chromosomes of tetraploid *A. cristatum* (Fig. 6). Comparison of the FISH patterns of two tetraploid *A. cristatum* accessions revealed that the hybridization signals of pAcpCR2 were stable, whereas the signals of pAcTRT1 were polymorphic among homologous chromosomes, similar to those of diploid *A. cristatum*. The differences of FISH signals between the two accessions were the different frequencies of the occurrence of pAcTRT1, for example, two out of four 2P chromosomes of Z559 displayed pAcTRT1 signals on the long arm, whereas only one of the four 2P chromosomes of Z589 showed pAcTRT1 signals on the long arm (Fig. 6).

## Discussion

### The utility of FISH for identifying *A. cristatum* chromosomes

The identification of chromosomes is an important step in the analysis of genomic relationships. FISH is advantageous for this process because it can identify chromosomes quickly and accurately. With the development of FISH probes, an increasing number of plant chromosomes can be discriminated by FISH, such as those in Arabidopsis, wheat, barley, maize, soybean, and rye^14,40–44^. Recently, the utilization of oligonucleotide probes has facilitated the identification of chromosomes in certain plant genomes^18–20,45–48^. However, *A. cristatum* lags behind other plant species in terms of the identification of chromosomes. In the present study, all seven pairs of *A. cristatum* chromosomes could be discriminated by FISH using pAcTRT1 and pAcpCR2 as probes, which facilitated the cytogenetic study of this species. Although studies^36,37^ have identified diploid *A. cristatum* chromosomes by FISH, studies of chromosomal variations of different original species and polyploids will require more probes due to the open pollination and different ploidy levels of *A. cristatum*.

FISH probes can be used to identify *A. cristatum* chromosomes in wheat-*A. cristatum* introgression lines. FISH has been widely used to detect alien chromosomes in wheat backgrounds, such as chromosomes of rye, *Dasypyrum breviaristatum*, and *Thinopyrum bessarabicum*^20,45,49^. In this study, we found that pAcTRT1 and pAcpCR2 enabled the identification of P genome chromosomes in wheat-*A. cristatum* derivative lines. The P genome chromosomes identified in the wheat-*A. cristatum* derivative lines in this study were the same as those in our previous studies^34,38,39^, indicating that the present data are reliable. The arrangement of diploid *A. cristatum* chromosomes in this study was determined through their correspondence to the P chromosomes in these introgression lines. Said *et al*.^36^ arranged the P genome chromosome via mapping wheat cDNAs on homoeologous chromosomes of *A. cristatum*. Both of these methods are performed based on the homoeology with wheat chromosomes. The distribution of 45S rDNA on diploid *A. cristatum* chromosomes were the same in both studies. The different distribution of pSc200 on diploid *A. cristatum* chromosomes between the two studies was probably due to the different diploid *A. cristatum* accessions used. Linc *et al*.^37^ also discriminated the chromosomes in diploid *A. cristatum*, but the order of chromosomes was not determined according to the homoeology with wheat. In addition, pAcpCR2 is a pericentromeric sequence specific to *A. cristatum* and potentially useful for deducing the translocated region of P genome chromosomes. The probes pAcTRT1 and pAcpCR2 may be utilized in other fields. The successful identification of diploid and tetraploid *A. cristatum* chromosomes in this study suggests that the probes could be applied to compare chromosomal structures among different *A. cristatum* species, similar to the study by Zhao *et al*.^50^.

Chromosome discrimination contributes to chromosome flow sorting. Giorgi *et al*.^6^ isolated each wheat chromosome based on the variations in the distribution and abundance of repetitive DNA sequences among chromosomes. Two rapid gene-cloning methods based on chromosome flow sorting, namely, ‘MutChromSeq’ and ‘targeted chromosome-based cloning via long-range assembly’ (TACCA), have been developed^51,52^. These methods may be valuable for cloning desirable genes transferred from *A. cristatum* to wheat. The identification of *A. cristatum* chromosomes in the present study is a key factor that will enable such cloning.

### *A. cristatum* chromosomal polymorphisms

Chromosomal structural polymorphisms can be found not only among species but also among different individuals within one population^53,54^. In this study, polymorphisms of the homologous chromosomes in *A. cristatum* were identified with FISH analysis using the repeat pAcTRT1. We first ruled out the possibility of technical artefacts based on the identical signal distributions of different FISH patterns from one individual plant. Although chromosomal structural polymorphisms also occur among different wheat cultivars from different regions^17,55^, there are differences between such polymorphisms and those in *A. cristatum*. The polymorphisms in *A. cristatum* occur naturally and among different individuals in one population, whereas the chromosomal structures among different individuals in one wheat cultivar are generally the same. This phenomenon can be attributed to the open pollination of *A. cristatum*. The genetic exchange and recombination between and within species led to chromosomal structural polymorphisms, and the distal regions were more actively involved than centromeric regions in rearrangements^56^. These features of polymorphisms were also found by Linc *et al*.^37^ and Said *et al*.^36^. In addition, slight differences were found between certain homologous chromosomes in one cell, such as 2P and 6P (Figs 1, 2). These differences may partially account for the production of six wheat-*A. cristatum* 6P addition lines^27^.

Chromosomal structural polymorphisms are the reason we collect wild resources at the population level and conserve individuals separately. The polymorphisms also result in differences in certain traits among individuals. Different types of individuals should be utilized according to the targets of wheat improvement. Therefore, the study of chromosomal polymorphisms will be helpful for the transfer of desirable genes from wild individuals to wheat.

## Methods

### Plant materials

Detailed information for all the materials used in this study is provided in Table 2. The materials included diploid *A. cristatum* Z1842 (2*n* = 2*x* = 14, PP, from Inner Mongolia, China), two tetraploid *A. cristatum* accessions Z559 (2*n* = 4*x* = 28, PPPP, from Xinjiang, China) and Z589 (2*n* = 4*x* = 28, PPPP, from Inner Mongolia, China), fourteen different wheat-*A. cristatum* disomic addition lines, and three derivative lines containing multiple alien P genome chromosomes. All fourteen addition lines (4844-12, 5113, 5114, 5106, II-26, II-5-1, II-4-2, 5038, 5043, II-21-2, II-21-6, II-9-3, II-7-1, and II-8-1) contain 42 wheat chromosomes and a pair of alien *A. cristatum* chromosomes. Wheat-*A. cristatum* derivative II-3-1 was a 1P (1A) disomic substitution and 2P disomic addition line. II-23-1 was a multiple 4P (4B), 7P (7A) substitution line with one pair of additional 2P chromosomes. II-11-1 was a wheat-*A. cristatum* 2P and 5P double disomic addition line. The P genome chromosomes in these lines were identified based on the homoeology with wheat in our previous studies^25,27–29,33,34,38,39^. These materials were stored or created by our laboratory.

**Table 2:** Materials used in this study.

Accession no.	Species	Genome	The number of chromosomes	References
Z1842	*A. cristatum* Gaertn.	PP	14	
Z559	*A. cristatum* Gaertn.	PPPP	28	
Z589	*A. cristatum* Gaertn.	PPPP	28	
4844-12^a^	*T. aestivum* L.	AABBDD + II6P	44	Wu *et al*.^25^; Han *et al*.^27^
5113^a^	*T. aestivum* L.	AABBDD + II6P	44	Han *et al*.^27^; Li *et al*.^28^
5114^a^	*T. aestivum* L.	AABBDD + II6P	44	Han *et al*.^27^
5106^a^	*T. aestivum* L.	AABBDD + II6P	44	Han *et al*.^27^
II-26^a^	*T. aestivum* L.	AABBDD + II6P	44	Han *et al*.^27^
II-9-3^a^	*T. aestivum* L.	AABBDD + II2P	44	Li *et al*.^29,35^
II-7-1^a^	*T. aestivum* L.	AABBDD + II2PS	44	Zhou *et al*.^38^
II-8-1^a^	*T. aestivum* L.	AABBDD + II2PS	44	Zhou *et al*.^38^
II-4-2^a^	*T. aestivum* L.	AABBDD + II7P	44	Zhou *et al*.^38^
II-5-1^a^	*T. aestivum* L.	AABBDD + II7P	44	Lu *et al*.^33^
5038^a^	*T. aestivum* L.	AABBDD + II7P	44	Zhou *et al*.^38^
5043^a^	*T. aestivum* L.	AABBDD + II7P	44	Zhou *et al*.^38^
II-21-2^a^	*T. aestivum* L.	AABBDD + II4P	44	Zhou *et al*.^38^
II-21-6^a^	*T. aestivum* L.	AABBDD + II4P	44	Zhou *et al*.^38^
II-3-1^b^	*T. aestivum* L.	AABBDD - II1A + II(1P + 2P)	44	Pan *et al*.^34^
II-23-1^c^	*T. aestivum* L.	AABBDD-II(4B + 7 A) + II(2P + 4P + 7P)	44	Chen *et al*.^39^
II-11-1^d^	*T. aestivum* L.	AABBDD + II(2P + 5P)	46	Zhou *et al*.^38^

^a^Wheat–*A. cristatum* addition lines; ^b^wheat-*A. cristatum* derivative II-3-1 was a 1P (1A) disomic substitution and 2P disomic addition line; ^c^wheat-*A. cristatum* derivative II-23-1 was a multiple 4P (4B), 7P (7A) substitution line with one pair of additional 2P chromosomes; ^d^wheat-*A. cristatum* derivative II-11-1 was a 2P and 5P disomic addition line.

### Acquisition of *A. cristatum-*specific probes

Two *A. cristatum-*specific probes (pAcTRT1 and pAcpCR2), which distribute on the telomeric and pericentromeric regions of *A. cristatum* chromosomes, were used as FISH probes. The probes were recovered from degenerate oligonucleotide primed-polymerase chain reaction (DOP-PCR) products of microdissected chromosome 6PS^11,12^. The two sequences were registered in NCBI GenBank with the accession numbers KP231286 and KX390711.

### Fluorescence ***in situ*** hybridization

Seeds were germinated on moistened filter paper in Petri dishes. Actively growing roots were removed from seedlings and subjected to nitrous oxide treatment for 2 h, fixed in 90% acetic acid for 8 minutes and stored in 70% v/v ethanol. Chromosome preparation and FISH were performed following the protocols described by Kato *et al*.^40^ and Han *et al*.^57^. Cytological observations were performed using a BX51 Olympus phase-contrast microscope (Olympus Corp., Tokyo, Japan). FISH with the DNA clones pAcTRT1 and pAcpCR2 was used to explore the clone distributions on P genome chromosomes in *A. cristatum* and wheat-*A. cristatum* derivative lines. The probes for 45S rDNA from wheat, rye tandem pSc200 repeat and *Aegilops tauschii* Coss. clone pAs1 were used to verify the order of the P genome chromosomes in diploid *A. cristatum*. The probes were labelled with Texas Red-5-dCTP or fluorescein-12-dUTP (PerkinElmer, Boston, MA, USA). The total volume of hybridization mixture per slide was 6 µl, containing 0.5 µl of 100 ng/µl probe, with the balance of the volume comprising 2 × SSC and 1 × TE. All the images were obtained using an Olympus AX80 fluorescence microscope (Olympus Corp., Tokyo, Japan) and processed with Adobe Photoshop CS 3.0 (Adobe, San Jose, CA, USA).

### Karyotype and idiogram for *A. cristatum* chromosomes

To observe interindividual chromosomal polymorphisms of diploid *A. cristatum*, five individual plants were selected randomly from population Z1842 of diploid *A*. *cristatum*. The chromosomes of 10 well-spread metaphase cells for each individual were used for karyotype analysis and FISH pattern analysis. Chromosome measurements were performed with Image J software (http://rsb.info.nih.gov/ij) and converted to relative lengths. Relative length was determined by dividing the length of a particular chromosome by the total length of chromosomes in the haploid set. The identification and construction of the idiogram for the P genome were based on the FISH pattern, chromosome length and arm ratio. The order of the P genome chromosomes was determined mainly according to the identification of added chromosomes in wheat-*A. cristatum* addition lines. The construction of the idiogram and the polymorphism analysis of FISH patterns were performed according to Wang *et al*.^54^.

## Supplementary information

Supplementary information
