# Supplementary material for: Identification of P genome chromosomes in Agropyron cristatum and wheat-A. cristatum derivative lines by FISH
Source: Sci Rep. 2019 Jul 4;9:9712. doi: 10.1038/s41598-019-46197-6 (PMC6609639; doi:10.1038/s41598-019-46197-6)
Supplement: Supplementary file 1 — Supplementary information [file 41598_2019_46197_MOESM1_ESM.pdf]

Supplementary information

Identification of P genome chromosomes in *Agropyron cristatum* and wheat-*A. cristatum* derivative lines by FISH

Haiming Han, Weihua Liu, Jinpeng Zhang, Shenghui Zhou, Xinming Yang, Xiuquan Li, Lihui Li*

Affiliation

National Key Facility for Crop Gene Resources and Genetic Improvement (NKCRI), Institute of Crop Sciences, Chinese Academy of Agricultural Sciences, Beijing 100081, China

*Corresponding author: E-mail: lilihui@caas.cn, Fax: +86-10-62189650

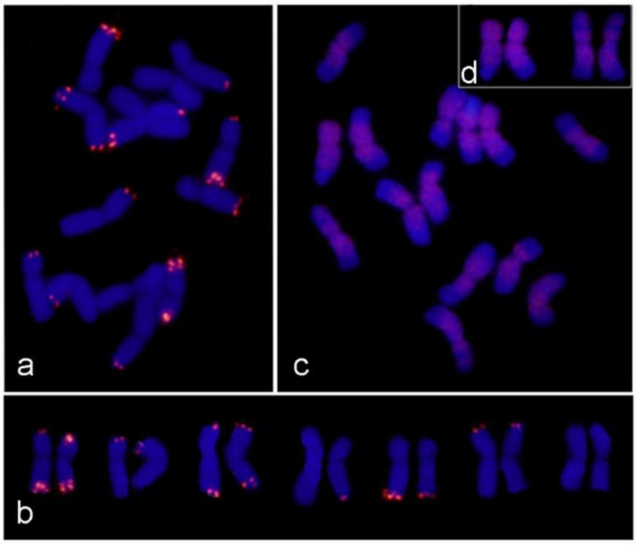

Supplementary Figure 1 Identification of diploid *A. cristatum* Z1842 chromosomes using pAcTRT1 (**a**) and pAcpCR2 (**c**) as probes. **a**, **c**: FISH patterns of two different individuals from Z1842; **b**: all the chromosomes were identified by pAcTRT1; **d**: there were two pairs of chromosomes identified by pAcpCR2.

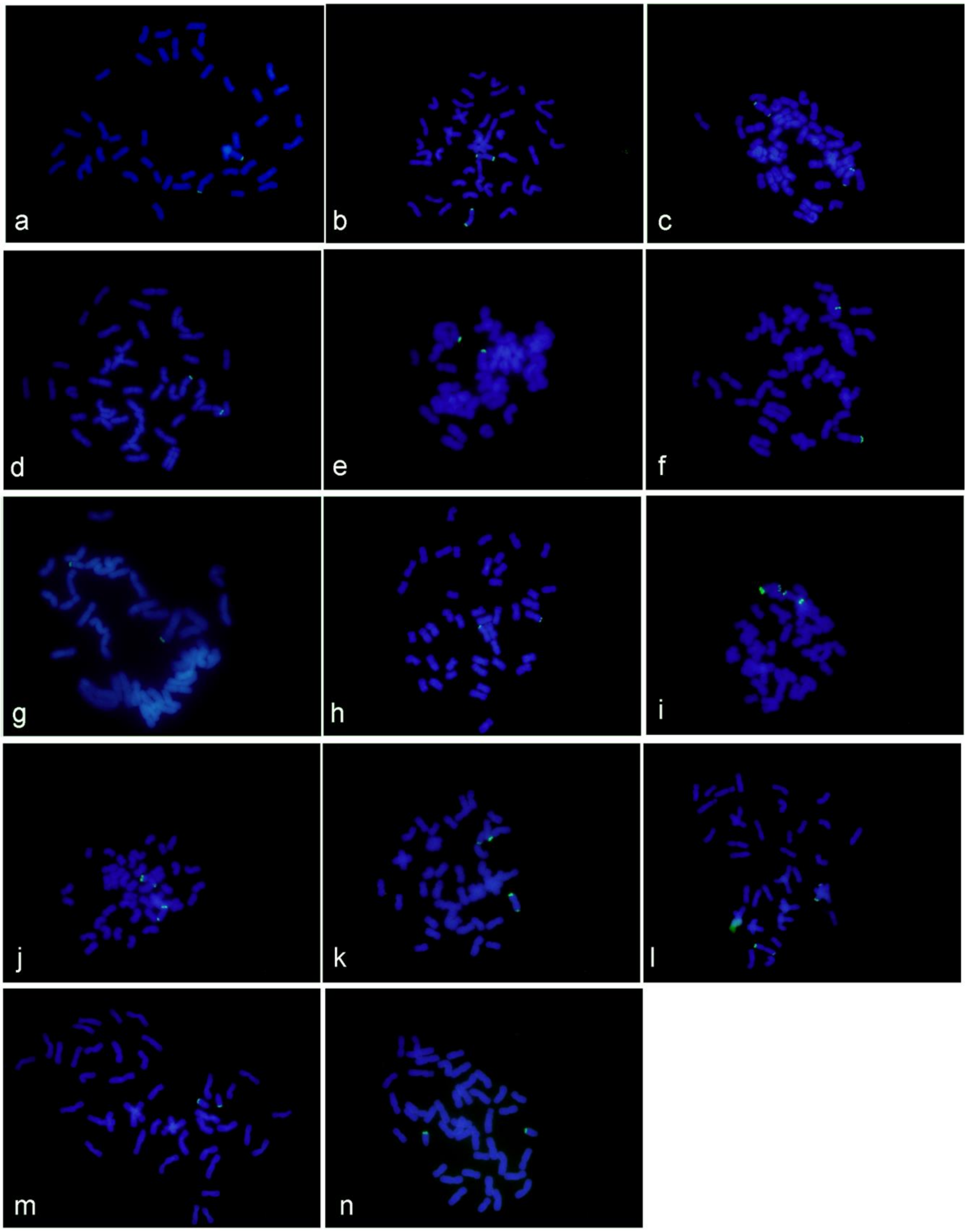

Supplementary Figure 2 FISH patterns of 14 wheat-*A. cristatum* addition lines using pAcTRT1 as the probe. **a**: II-9-3, **b**: II-21-2, **c**: II-21-6, **d**: 4844-12, **e**: 5113, **f**: 5114, **g**: 5106, **h**: II-26, **i**: 5038, **j**: 5043, **k**: II-4-2, **l**: II-5-1, **m**: II-7-1, **n**: II-8-1.

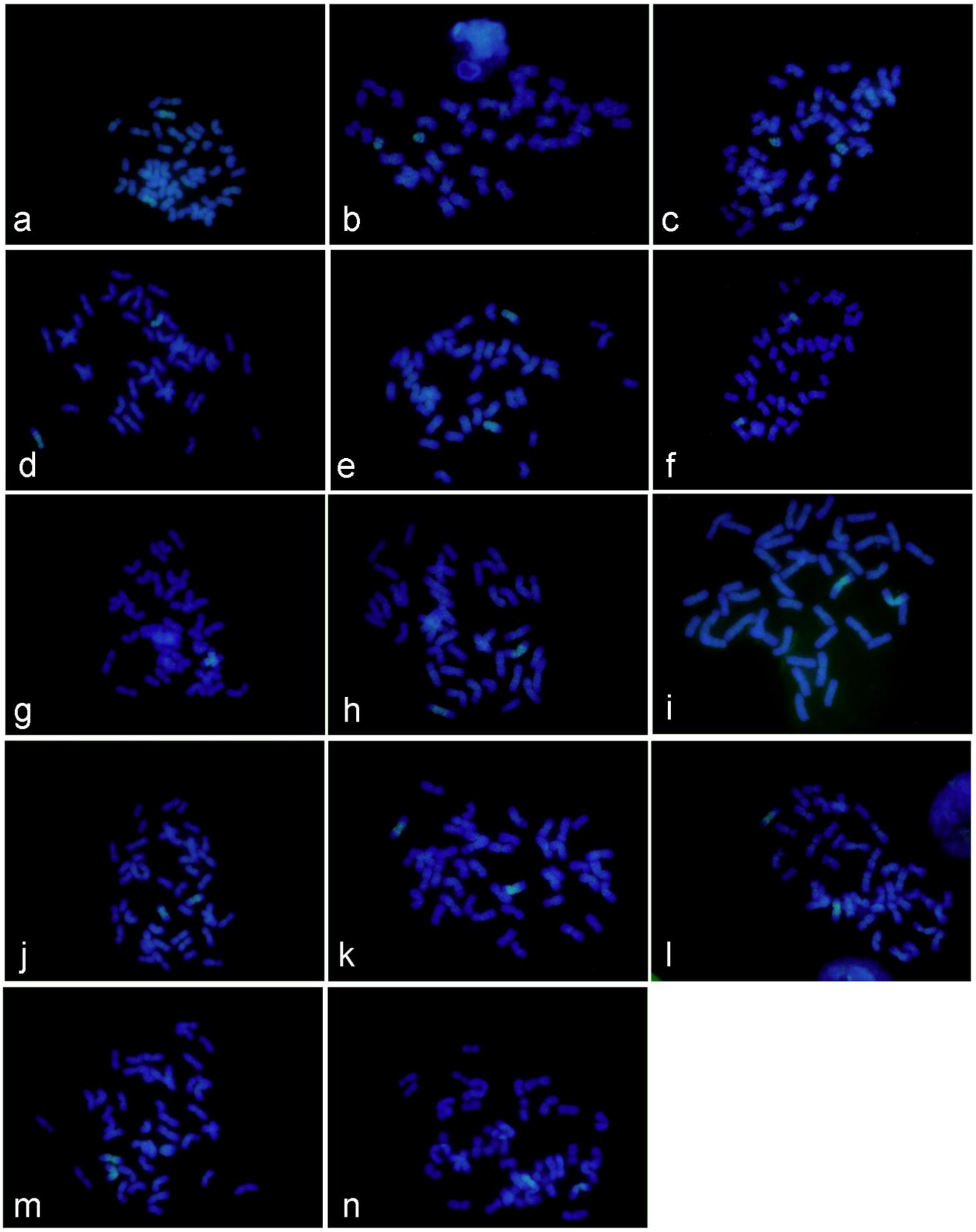

Supplementary Figure 3 FISH patterns of 14 wheat-*A. cristatum* addition lines using pAcpCR2 as the probe. **a**: II-9-3, **b**: II-7-1, **c**: II-8-1, **d**: II-21-2, **e**: II-21-6, **f**: 5113, **g**: 4844-12, **h**: 5114, **i**: 5106, **j**: II-26, **k**: 5038, **l**: 5043, **m**: II-4-2, **n**: II-5-1.

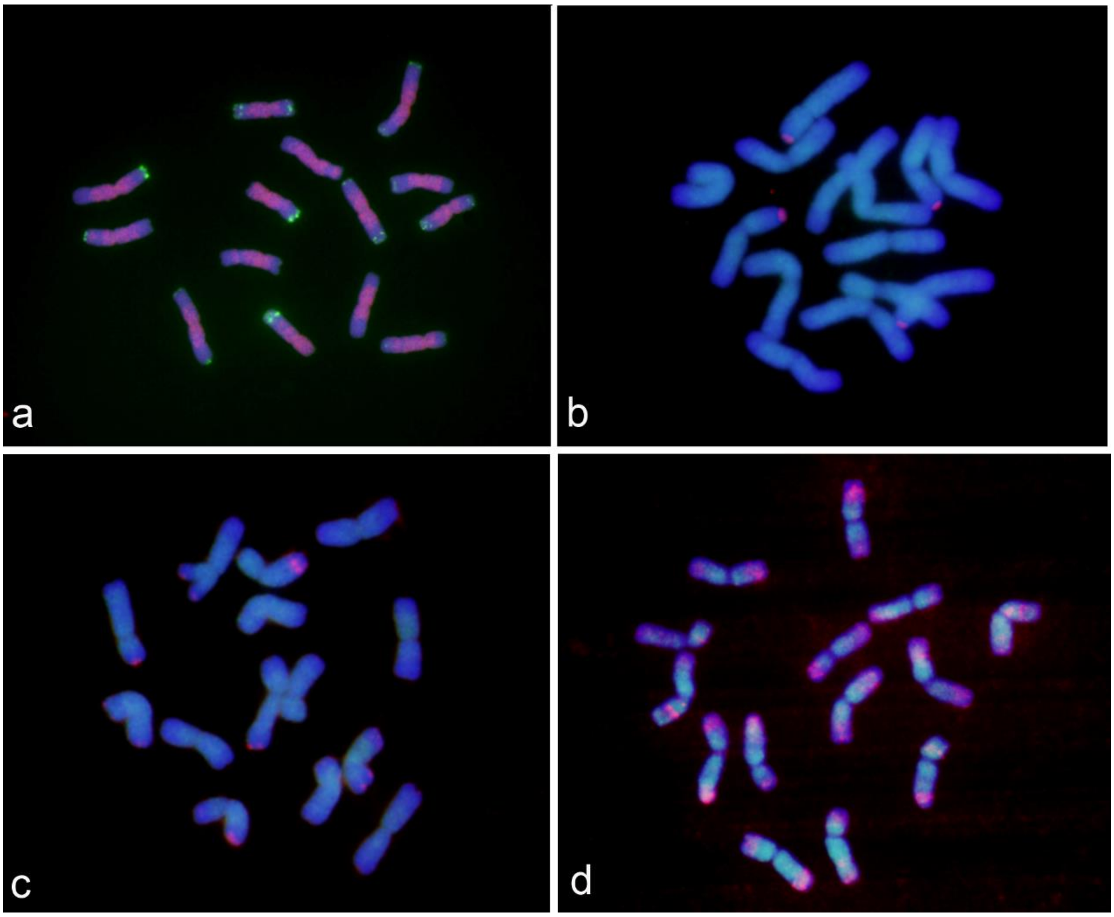

Supplementary Figure 4 FISH patterns of diploid *A. cristatum* Z1842. **a**: The green signals represent the probe pAcTRT1, and the red signals represent the probe pAcpCR2, **b**:The red signals represent the probe 45rDNA, and the green signals represent the probe pAcpCR2, **c**:The red signals represent the probe pSc200, and the green signals represent the probe pAcpCR2, **d**:The red signals represent the probe pAs1, and the green signals represent the probe pAcpCR2.
